# Interleukin‐22 in urinary tract disease – new experimental directions

**DOI:** 10.1002/cti2.1143

**Published:** 2020-06-07

**Authors:** Molly A Ingersoll, Malcolm R Starkey

**Affiliations:** ^1^ Department of Immunology Institut Pasteur Paris France; ^2^ INSERM U1223 Paris France; ^3^ Department of Immunology and Pathology Central Clinical School Monash University Melbourne VIC Australia; ^4^ Priority Research Centre GrowUpWell Faculty of Health and Medicine The University of Newcastle Callaghan NSW Australia

## Abstract

Interleukin (IL)‐22 is expressed by immune cells in the urinary tract and IL‐22 receptor is expressed in urothelium and renal tubule cells. IL‐22 can be specifically targeted in the urinary tract or conditionally depleted in mice and targeted therapeutically in humans.

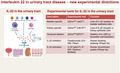

Interleukin (IL)‐22 is a cytokine produced by immune cells with bimodal functionality depending on the tissue it is expressed in and the context of its expression. IL‐22 has antimicrobial properties and can limit bacterial infections but also drive pro‐inflammatory responses that may have deleterious consequences for disease progression. Conversely, IL‐22 can also promote epithelial cell repair, regeneration and restoration of mucosal barrier function. IL‐22 may also alter the microbiota, which has broad implications for the maintenance of homeostasis at mucosal surfaces colonised by commensal microorganisms, such as the gastrointestinal or the urinary tract. A critical limitation in the exploration of IL‐22 function, at sites such as in the urinary tract, is a lack of appropriate experimental tools. Studies using newly available, genetically modified reporter mice have led to the identification of the cellular source and location of IL‐22, while Cre‐lox expressing transgenic mice permit specific targeting of IL‐22 in the urinary tract. With a greater understanding of the role of IL‐22 in the urinary tract, repurposing of emerging novel human immunotherapies that either inhibit or activate IL‐22 may create new opportunities for the treatment of urinary tract diseases.

## IL‐22 at mucosal surfaces

**Figure 1:**
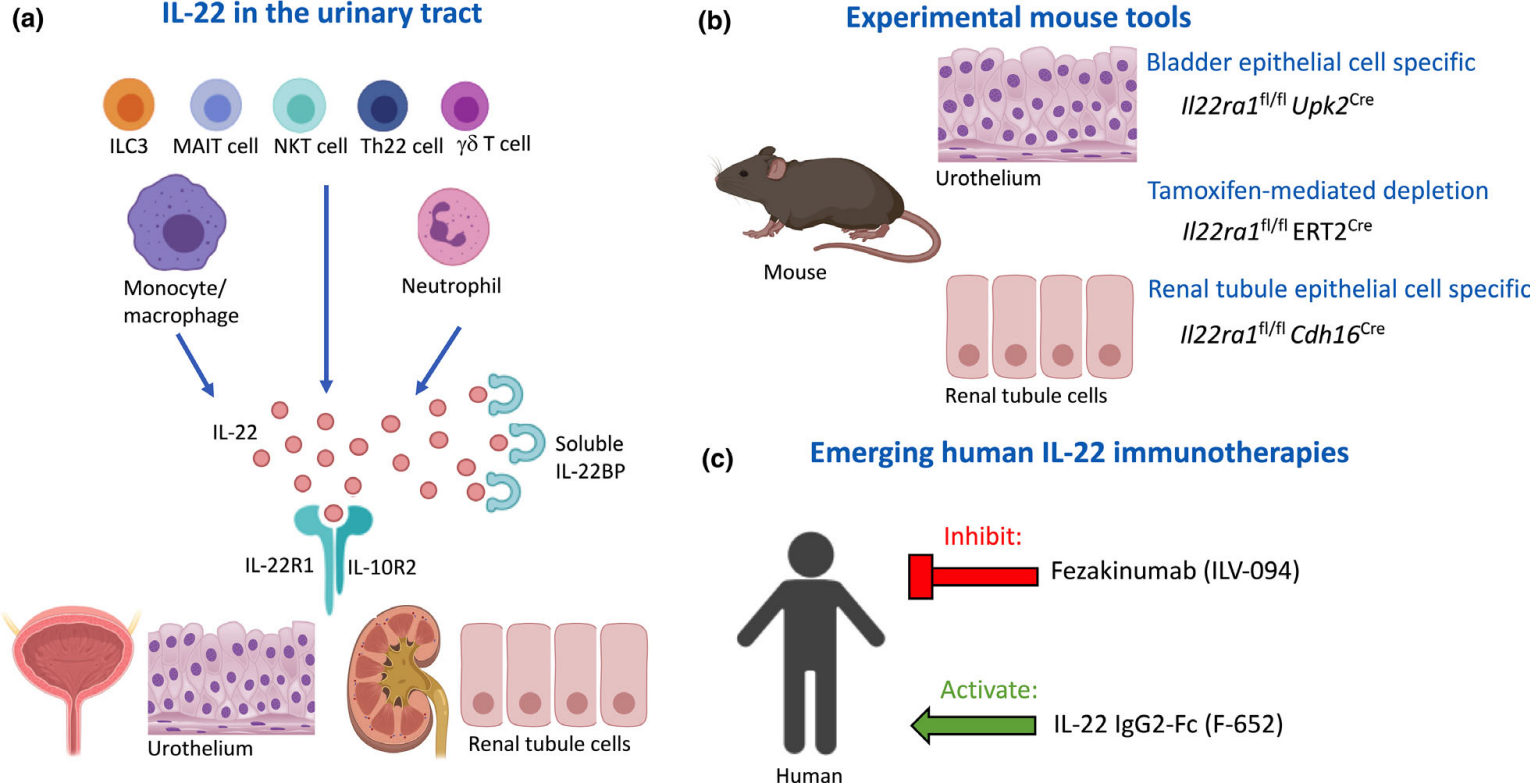
IL‐22 and IL‐22 receptors in the urinary tract, new tools to assess their function *in vivo* and emerging immunotherapies. **(a)** IL‐22 is produced by several immune cell subsets including group 3 innate lymphoid cells (ILC3), mucosal‐associated invariant T cells (MAIT), natural killer T cells (NKT), T helper 22 cells (Th22), γδT cells, macrophages and neutrophils. IL‐22 signals through a membrane‐bound heterodimer complex consisting of IL‐22R1 and IL‐10R2 expressed on urothelium and renal tubule epithelial cells in the bladder and kidney, respectively. The binding of IL‐22 to this membrane‐bound receptor complex activates downstream signalling pathways to induce antimicrobial, pro‐inflammatory or tissue repair/regenerative responses. IL‐22 activity is negatively regulated by the soluble high‐affinity receptor IL‐22BP. **(b)** Experimental mouse tools that specifically target IL‐22 receptor in the bladder and kidney epithelium, respectively. Conditional depletion of IL‐22 receptor at specific time points can be achieved by tamoxifen administration. These tools may enable future detailed mechanistic studies for multiple urinary tract disease models. **(c)** Emerging immunotherapies that target IL‐22 in humans.

IL‐22 is an IL‐10 family cytokine produced mainly by immune cells of the lymphoid lineage including group 3 innate lymphoid cells (ILC3), mucosal‐associated invariant T cells (MAIT), natural killer T cells (NKT), T helper 22 cells (Th22) and γδT cells^1^ (Figure 1a). However, macrophages or neutrophils can also produce IL‐22 under certain circumstances, such as in a model of dextran sodium sulphate‐induced colitis, in which IL‐22‐expressing neutrophils contribute to epithelial repair in the colon.^2^

IL‐22 signals through the IL‐22 receptor (IL‐22R), which is composed of two heterodimeric subunits, IL‐22R1 (encoded by *Il22ra1* in mice) and IL‐10R2.^1^ IL‐22 has a high affinity for IL‐22R1, and while it has no affinity for IL‐10R2, the IL‐22‐IL‐22R1 complex has a strong binding affinity for the IL‐10R2 subunit (Figure 1a). IL‐22R1 expression is restricted to renal tubular epithelial cells in mice.^3^ Its expression in the bladder epithelium is unreported, but it is typically expressed in epithelial surfaces.

Indeed, IL‐22 primarily targets non‐haematopoietic epithelial cells at mucosal barrier sites (e.g. lung, skin, gastrointestinal tract), where it promotes proliferation of epithelial cells and tissue regeneration after injury.^2^ IL‐22 regulates host microbial defences through induction of antimicrobial peptides, such as the Reg family of proteins during *Citrobacter rodentium* infection.^4^ Studies in the gastrointestinal tract demonstrate that IL‐22, expressed by ILCs, helps to maintain containment of commensal bacterial strains, and in the absence of these cells, specific microbes can breach the gastrointestinal barrier.^5^ Given that IL‐22 is produced at sites of inflammation, it may either mediate a physiologic response to repair local tissue damage or it may contribute to inflammatory lesions.^1^ Therefore, caution is needed when interpreting the impact of IL‐22 in disease models and there is a clear need for organ‐ and tissue‐specific tools to accurately assess its function.

## IL‐22 in the kidney

The role of IL‐22 in the kidney is reasonably well studied (reviewed in Weidenbusch *et al.* ^6^). Infiltrating immune cells secrete IL‐22, inducing progressive kidney remodelling following unilateral ureteral obstruction, which augments renal tubular epithelial integrity and epithelial barrier function.^7^ IL‐22 accelerates kidney regeneration and ameliorates renal ischaemia–reperfusion injury,^3^, ^8^ suggesting, overall, that IL‐22 function in the kidney is most likely tissue‐protective and that restoring optimal IL‐22 function may be beneficial in certain kidney diseases. Interestingly, however, endogenous IL‐22 does not play a role in glomerulonephritis,^9^ highlighting that IL‐22 function in the kidney is disease‐dependent.

## New experimental directions for functional characterisation of IL‐22 in the urinary tract

There are various experimental mouse tools that can facilitate a detailed mechanistic understanding of IL‐22 function *in vivo*. IL‐22 reporter mice (e.g. *Il22* ^td‐tomato^ mice^10^) allow for quantification of endogenous IL‐22^+^ immune cells in the urinary tract and other tissues without *ex vivo* cell stimulation used in intracellular cytokine staining procedures.^10^ This is a strategic advantage as it gives a readout of IL‐22 expression in tissue and eliminates the caveats of artificial stimulation *ex vivo*. Reporter mice also bypass reliance upon antibody specificity for the intracellular cytokine, although nonspecific fluorescent leakage of reporter mice is still a concern. In addition to identifying the cells producing IL‐22, localisation of cytokine‐producing cells is possible using 3‐dimensional imaging of optically transparent IL‐22 reporter mouse tissue in high resolution with 2‐photon fluorescent microscopy.^11^ Finally, reporter mice can be used for real‐time *in vivo* imaging and tracking of IL‐22^+^ cell migration into organs of the urinary tract.

IL‐22‐deficient and IL‐22 receptor‐deficient mice are key to providing information on the impact of a lack of IL‐22 signalling systemically. However, because of the complex multifaceted and context‐specific nature of IL‐22 signalling, systemic deletion will likely provide confounding results. IL‐22 is also critical for the maintenance and regulation of the gastrointestinal microbiota,^4^, ^5^ which is known to impact multiple diseases of the urinary tract both clinically and experimentally. To improve future experimental design and limit unintentional off‐target effects of IL‐22 systemically and on the microbiota, mice that specifically lack the IL‐22 receptor in bladder or kidney epithelial cells should be created. This can be achieved by crossing IL‐22 receptor‐floxed (^fl/fl^) mice (*Il22ra1* ^fl/fl^, The Jackson Laboratory, USA) with the mouse uroplakin 2 (Upk2) promoter driving Cre recombinase (^Cre^) expression (*Upk2* ^Cre^, The Jackson Laboratory) in the bladder urothelium or by crossing the *Il22ra1* ^fl/fl^ mouse with Cre recombinase under the control of the mouse cadherin 16 (Cdh16) promoter (*Cdh16* ^Cre^, The Jackson Laboratory) resulting in loss of IL‐22 receptor expression in the renal tubules of adult mice (Figure 1b). *Il22ra1* ^fl/fl^ mice could also be crossed with tamoxifen‐induced (ERT2)^Cre^ mice to control when IL‐22 is disrupted in a given urinary tract disease model, with the caveat that tamoxifen‐mediated deletion is not restricted solely to the urinary tract.

## Emerging IL‐22 immunotherapies – potential for the treatment of urinary tract diseases

There are novel human immunotherapies in the clinical trial that either inhibit or enhance human IL‐22 activity (Figure 1c). Clinical safety and pharmacokinetic studies are completed for both of these therapies, and both are in phase IIa trials for human diseases.^12^, ^13^ Fezakinumab (ILV‐094, Pfizer) is a human anti‐IL‐22 monoclonal antibody that blocks IL‐22 and was developed for the treatment of moderate‐to‐severe atopic dermatitis.^12^ IL‐22 IgG2‐Fc (F‐652, Generon BioMed) is a human recombinant IL‐22 developed to promote IL‐22‐mediated tissue repair and regeneration in the context of graft‐*vs*‐host disease and alcoholic hepatitis.^13^ To facilitate the translation of emerging immunotherapies targeting IL‐22 in human urinary tract diseases, the critical first step will be to provide proof‐of‐principal studies in experimental mouse models of urinary tract disease, using tools that restrict the manipulation of IL‐22 specifically to the urinary tract. In this way, it will be apparent whether the addition or inhibition of IL‐22 is beneficial to the host response to urinary tract diseases.

## Conclusion

Without a clearly defined role for IL‐22 in the urinary tract using experimental approaches, it will be difficult to justify whether inhibition or enhancement of IL‐22 function in humans with urinary tract disease will be of therapeutic benefit. Strategies to specifically target these emerging immunotherapies to the urinary tract will be required as targeting IL‐22 systemically may have unwanted off‐target effects on other systems; however, the bladder, for example, is uniquely suited for local delivery *via* catheterisation. Careful consideration of the impact of IL‐22 therapies on the microbiota and its subsequent impact on urinary tract disease will also need to be carefully considered in future clinical trial design.

## Conflict of interest

The authors declare no conflict of interest.

## Author contributions

**Molly A Ingersoll:** Conceptualization; Writing‐original draft; Writing‐review & editing. **Malcolm R Starkey:** Conceptualization; Writing‐original draft; Writing‐review & editing.
